# The combined prognostic model of copper-dependent to predict the prognosis of pancreatic cancer

**DOI:** 10.3389/fgene.2022.978988

**Published:** 2022-08-10

**Authors:** Xiao Guan, Na Lu, Jianping Zhang

**Affiliations:** Department of General Surgery, The Second Affiliated Hospital of Nanjing Medical University, Nanjing Medical University, Nanjing, China

**Keywords:** copper-dependent genes, pancreatic cancer, immune infiltration, prognosis, bioinformatics

## Abstract

**Purpose:** To assess the prognostic value of copper-dependent genes, copper-dependent-related genes (CDRG), and CDRG-associated immune-infiltrating cells (CIC) for pancreatic cancer.

**Methods:** CDRG were obtained by single-cell analysis of the GSE156405 dataset in the Gene Expression Omnibus (GEO) database. In a ratio of 7:3, we randomly divided the Cancer Genome Atlas (TCGA) cohort into a training cohort and a test cohort. Tumor samples from the GSE62452 dataset were used as the validation cohort. CIBERSORT was used to obtain the immune cell infiltration. We identified the prognostic CDRG and CIC by Cox regression and the least absolute selection operator (LASSO) method. The clinical significance of these prognostic models was assessed using survival analysis, immunological microenvironment analysis, and drug sensitivity analysis.

**Results:** 536 CDRG were obtained by single-cell sequencing analysis. We discovered that elevated LIPT1 expression was associated with a worse prognosis in pancreatic cancer patients. EPS8, CASC8, TATDN1, NT5E, and LDHA comprised the CDRG-based prognostic model. High infiltration of Macrophages.M2 in pancreatic cancer patients results in poor survival. The combined prognostic model showed great predictive performance, with the area under the curve (AUC) values being basically between 0.7 and 0.9 in all three cohorts.

**Conclusion:** We found a cohort of CDRG and CIC in patients with pancreatic cancer. The combined prognostic model provided new insights into the prognosis and treatment of pancreatic cancer.

## Introduction

Pancreatic cancer has a high mortality rate (Wei and Hackert, 2021). The number of new cases and deaths from pancreatic cancer is approximately the same in 2020 (Sung et al., 2021). Pancreatic cancer has the characteristics of high heterogeneity, difficult early diagnosis, limited efficacy, and poor prognosis (Park et al., 2021). Patients with pancreatic cancer are already at an advanced stage when diagnosing (Kamisawa et al., 2016). Surgery is still the most effective way to treat pancreatic cancer (Vincent et al., 2011). Despite improvements in treatment, pancreatic cancer has a 5-years survival rate of less than 10% (Rawla et al., 2019). Many patients eventually relapse after surgery (Siegel et al., 2014). Emerging evidence suggests that targeted therapy based on genetic testing may provide a viable treatment option for overcoming the limitations of pancreatic cancer treatment (Dókus et al., 2020). However, the clinical application of targeted therapy based on genetic testing is very limited due to tumor heterogeneity and its complex molecular subtypes (Hosein et al., 2020; Wang et al., 2021a; Giuliani et al., 2021). Therefore, new prognostic biomarkers and therapeutic targets are urgently needed. This will help clinicians to timely and accurately predict patient prognosis and develop personalized treatment plans.

Multiple cells in pancreatic cancer now can be studied accurately due to the advances in single-cell sequencing, which is a strong method for characterizing diverse cell types and has been used to study a variety of cancers (Treutlein et al., 2016; Ziegenhain et al., 2017). At the same time, through cell clustering and annotation, we can better understand the cellular differentiation and immune mechanisms of pancreatic cancer (Hwang et al., 2021).

Defects in the execution of cell death by tumor cells are one of the main reasons for their resistance to therapy (Hassannia et al., 2019). As a form of regulated cell death, copper-dependent death occurs through the direct binding of copper to fatty acylation components of the tricarboxylic acid cycle (Tsvetkov et al., 2022). Copper has two roles in carcinogenesis: it promotes tumor development while also causing redox stress in cancer cells (Maung et al., 2021). Copper is also used to treat cancer as a medication component and as a regulator of drug sensitivity and absorption (Maung et al., 2021). The study by *Yu et al.* confirmed that copper deficiency may be a novel approach to the treatment of pancreatic cancer (Yu et al., 2019a).

Tumor cells make up a minor portion of pancreatic cancer tissue, with the extracellular matrix accounting for the majority of the rest (Sherman et al., 2014). Pancreatic cancer has a broad immunosuppressive microenvironment that promotes cancer cell proliferation by directly suppressing antitumor immunity or evading immune surveillance (Ren et al., 2018; Zhang et al., 2021). Though in pancreatic cancer patients, a variety of immunotherapies have been explored, the majority of them have not been satisfactory (Looi et al., 2019; Leinwand and Miller, 2020).

Herein, we first identified CDRG in pancreatic cancer by single-cell sequencing analysis. Based on these CDRG, we also identified CIC in pancreatic cancer patients. Based on the above analysis results, we constructed a combined predictive model for pancreatic cancer patients that can effectively predict their prognosis. This study informed the treatment strategy for pancreatic cancer.

## Material and Methods

### Data collection

The TCGA database (TCGA-PAAD; URL: https://portal.gdc.cancer.gov/) was used to get the transcriptome and clinical data. The workflow type we used was Counts. The GEO database (https://www.ncbi.nlm.nih.gov/geo/) was used to obtain the pancreatic cancer single-cell sequencing dataset GSE156405. We screened the data from 4 patients. We also downloaded a microarray gene expression profile dataset GSE62452. All data were log2 transformed. 10 copper-dependent genes (Negative hits: MTF1, GLS, CDKN2A; Positive hits: FDX1, LIAS, LIPT1, DLD, DLAT, PDHA1, PDHB) were obtained from the study by *Tsvetkov et al.* (Tsvetkov et al., 2022).

### Data processing of the GSE156405

First, we performed quality control on the data. The data of 4 patients were obtained from it. Cells with less than 1% of mitochondrial genes, ribosomal genes, and erythrocyte genes and cells with between 500 and 4,000 total genes were retained. Genes expressed in at least 3 cells were kept. We identified the 6,000 most fluctuating genes based on their degrees of fluctuation across all samples. The “CellCycleScoring” function was used to judge the selected cell cycle, and the “ScaleData” function was used to remove the influence created by the cell cycle. The LogNormalize method was used to normalize and integrate the samples. After the data was corrected, principal component analysis was used for dimensionality reduction of the data, and TSNE was used for cluster analysis. We used the “SingleR” package to annotate cell kinds. We download the singler database, import “ref Human all.Rdata” into the environment, then use the singler method to define cell subsets. The “PercentageFeatureSet” function was used to calculate the proportion of copper-dependent genes in each cell after importing them. We classified the cells as low_cuproptosis or high_cuproptosis based on the median ratio of copper-dependent genes. Then, we use the “FindMarkers” function to identify the genes that differ between low_cuproptosis and high_cuproptosis cells, and we selected the genes to screen out the genes whose *p*-value is less than 0.05. These genes were classified as copper-dependent-related genes (CDRG).

### Data processing of the cancer genome atlas

First, the data downloaded were preprocessed and combined using the Perl language to access the count file. The gene symbol was also transformed with Perl. Then, the corresponding gene expression was acquired by matching the transcriptome data from TCGA with CDRG. We excluded patients with incomplete clinical data and those with 0 days of follow-up. We matched the CDRG expression data to the survival data, ran a univariate COX analysis, and filtered out prognostically significant genes with a *p*-value less than 0.05. We used the “caret” package to randomly split the matched cohort into a training cohort and a test cohort in a 7:3 ratio.

### Data processing of the GSE62452

GSE62452 includes 69 tumor samples and 61 non-tumor samples. After excluding non-tumor samples. We used this dataset as a validation cohort.

### Weighted Co-Expression network analysis

WGCNA analysis is a systems biology approach for characterizing patterns of genetic association between different samples (Langfelder and Horvath, 2008). It can be used to identify highly covalent gene sets and to identify candidate biomarker genes or therapeutic targets based on the interconnection of each gene set and the association between the gene set and the phenotype. We used the “WGCAN” package to generate the CDRG module. From there, we selected the modules that were relevant to survival time and survival status for subsequent analysis.

### Prognostic model based on copper-dependent-related genes

First, we matched GRCD expression data to survival data and performed the univariate COX analysis (*p* < 0.01). The prognostic genes were shown in the forest diagram. Then, we further selected GRCD with prognostic significance using the LASSO regression method. The prognostic model was built and the risk score of each patient was calculated. We divided BC patients into high- and low-risk groups based on median score. Between the two, we used clinical correlation heat maps to analyze differences in clinical characteristics and to examine differences in patient prognosis. The survival differences were then verified. Univariate and multivariate cox analyses were then performed to analyze risk scores and different clinical information.

### Validation and evaluation of the copper-dependent-related genes-based prognostic model.

The risk score for each sample in the test cohort was calculated using the model formula. After that, in training and test cohorts, survival analyses were carried out to see if there were any variations in prognosis between the two groups. Simultaneously, we plotted the distributions of samples between the two groups to determine the effectiveness of differentiating patients based on risk values. The expression of model genes was compared using heatmaps. Subsequently, we plotted the time-dependent receiver operating characteristic (ROC) plots and calculated the area under the curve (AUC) to validate the predictive power of the constructed prognostic model. In the validation cohort, we calculated the risk score for each patient. Next, survival analyses were performed. The model’s accuracy was assessed by the ROC curve.

### Functional enrichment analysis

We performed the Gene Ontology (GO) analysis and the Kyoto Encyclopedia of Genes and Genomes (KEGG) pathway analysis by the “clusterProfiler” package and the results were kept if the *p*-value < 0.05. The bar charts were used to represent the results of the analysis.

### Immunoassay analysis

To explore if there’s a link between our model and the level of tumor infiltration, we devised two ways to visualize our data: the immune infiltration heatmap and the correlation map. The tumor infiltration methods we used were CIBERSORT and XCELL (Newman et al., 2015; Aran et al., 2017). We found a list of immune checkpoint-related genes in the literature. The analysis results were displayed using boxplots.

### Identify copper-dependent genes associated with prognosis

We matched transcriptome data from TCGA to copper-dependent genes to obtain corresponding gene expression and excluded patients with incomplete clinical data and 0-days follow-up. Then, We matched copper-dependent gene expression data with survival data and performed the univariate COX analysis (*p* < 0.05) to obtain prognostic copper-dependent genes.

### Identification of copper-dependent-related genes-associated immune-infiltrating cells (CIC)

The composition of 22 immune cells in pancreatic cancer was obtained by CIBERSORT analysis (Newman et al., 2015). Rainbow graphs were used to show the proportion of different immune cells in pancreatic cancer in each sample. Box plots were used to show variations in immune cell infiltration between high and low-risk groups. Immune cells with *p* less than 0.05 were defined as CDRG-associated immune cells. We matched CDRG-dependent immune cell infiltration results with survival data for further selection by LASSO regression. The screened cells are used for subsequent analysis.

### Combined prognostic model construction and validation

Multivariate Cox regression was applied to build the combined prognostic model in the training cohort from the risk score, the expression of the selected copper-dependent genes, and the infiltration results of CIC. Correlation coefficients were calculated by using multivariate Cox regression. To test the clinical impact of the combined model in the three cohorts, we drew survival curves and ROC curves.

### Drug sensitivity analysis

We used the expression matrix and drug processing information from the Cancer Genome Project (CGP, https://www.cancerrxgene.org/) to obtain the drugs associated with the combined model using the “pRROpheticPredict” function (Geeleher et al., 2014).

## Results

### Analysis of the gene expression omnibus dataset

**FIGURE 1:**
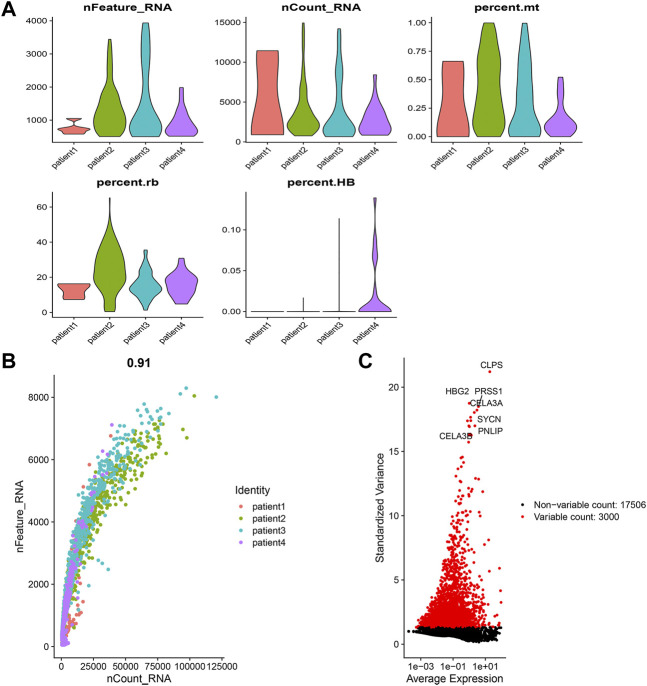
Single-cell sequencing analysis. **(A)** Integration and cluster analysis. We can find the distribution of these 14 clusters **(B)** Distribution of low-copper-dependent cells and high-copper-dependent cells. We found that the distribution of low_cuproptosis cells and high_cuproptosis cells in each cell cluster was relatively uniform. **(C)** WGCNA showed MEgrey was linked to the survival status.

The gene expression levels of each cell in the 4 samples ranged from 500 to 4,000, with a relatively uniform distribution. At the same time, we found that the percentage of mitochondrial genes was less than 1%, and the percentage of erythrocyte genes was basically less than 0.1% (Supplementary Figure S1A). Cells were evenly distributed among the 4 samples. The number of genes and their expression levels are positively correlated with a correlation coefficient of 0.91 (Supplementary Figure S1B). From all genes, we chose 3,000 hypervariable genes, which were highlighted in red and we also marked the top 10 genes (Supplementary Figure S1C). Then we integrated the 4 samples. The results showed that the integration could be used for subsequent analysis. After PCA dimensionality reduction, using the TSNE clustering technique, we divided all cells into 14 groups (Figure 1A). Then after using the “PercentageFeatureSet” function to input 10 copper-dependent genes, the proportion of them in each cell was obtained. According to the median ratio of copper-dependent genes, we divided the cells into low_cuproptosis and high_cuproptosis cells. The distribution of low_cuproptosis cells and high_cuproptosis cells in each cell cluster was relatively uniform (Figure 1B). Finally, between the two groups, we analyzed the differentially expressed genes and identified 536 CDRG.

### Weighted Co-Expression network analysis

WGCNA analysis of samples from the TCGA cohort identified gene modules related to clinical characteristics. We obtained 12 non-grey modules and screened out gene modules with *p* < 0.05 (Figure 1C). WGCNA analysis showed MEgrey was linked to the survival status. We selected it for subsequent analysis.

### Construction and evaluation of the copper-dependent-related genes-based prognostic model

**FIGURE 2:**
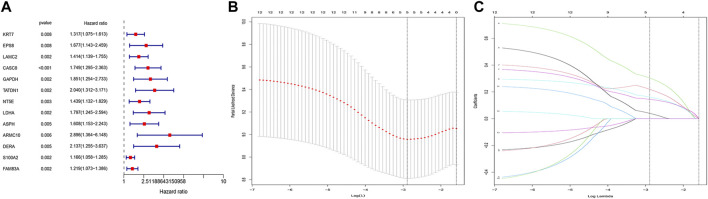
CDRG-based prognostic model construction. **(A)** Univariate COX analysis. There are 13 high-risk CDRGs, including KRT7, EPS8, LAMC2, CASC8, GAPDH, TATDN1, NT5E, LDHA, ASPH, ARMC10, DERA, S100A2, and FAM83A. **(B,C)** LASSO regression analysis. We performed LASSO regression analysis on these 13 genes. We finally obtained 5 modeling genes, which were EPS8, CASC8, TATDN1, NT5E, and LDHA. Risk score = EPS8*0.21942004 + CASC8*0.30361292 + TATDN1*0.16143689 + NT5E*0.14433129 + LDHA*0.03993997. We then divided the sample into high-risk groups and low-risk values based on the median risk score.

After matching transcriptomic data from TCGA and CDRG, we matched CDRG expression data with survival data and performed independent prognostic analysis, resulting in 13 genes with prognostic significance in the training cohort. Figure 2A showed the CDRG associated with prognosis. Then we performed Lasso regression analysis and screened 5 CDRG genes finally (Figures 2B,C). Based on these results, we constructed the prognostic model. The prognostic model was calculated as follows: risk score = EPS8*0.21942004 + CASC8*0.30361292 + TATDN1*0.16143689 + NT5E*0.14433129 + LDHA*0.03993997. At the same time, we calculated and recorded the risk score for each patient.

**FIGURE 3:**
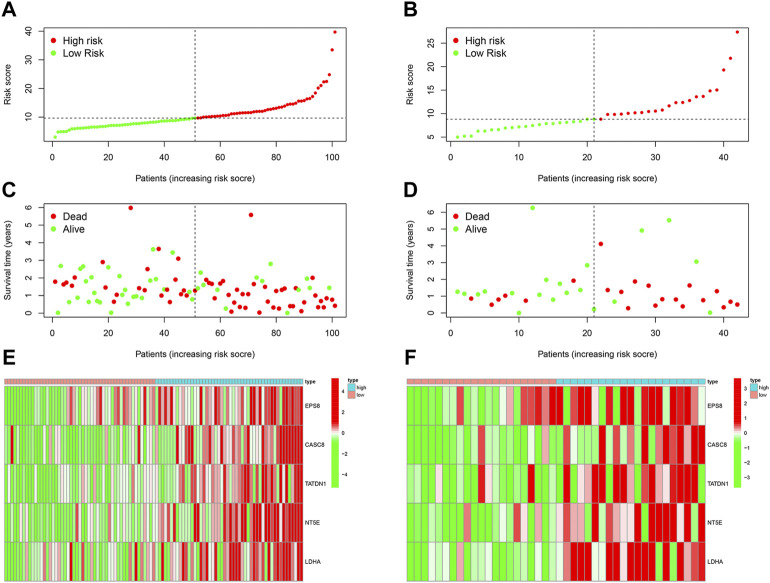
Evaluation of CDRG-based prognostic model. **(A,B)** We analyzed the distribution of gene expression and patient survival in the models between the high—and low-risk groups in training and test cohorts. **(C,D)** We found that with the increase in risk value, the proportion of BC patients who died increased. **(E,F)** Moreover, we found that genes EPS8, CASC8, TATDN1, NT5E, and LDHA were highly expressed in the low-risk group.

**FIGURE 4:**
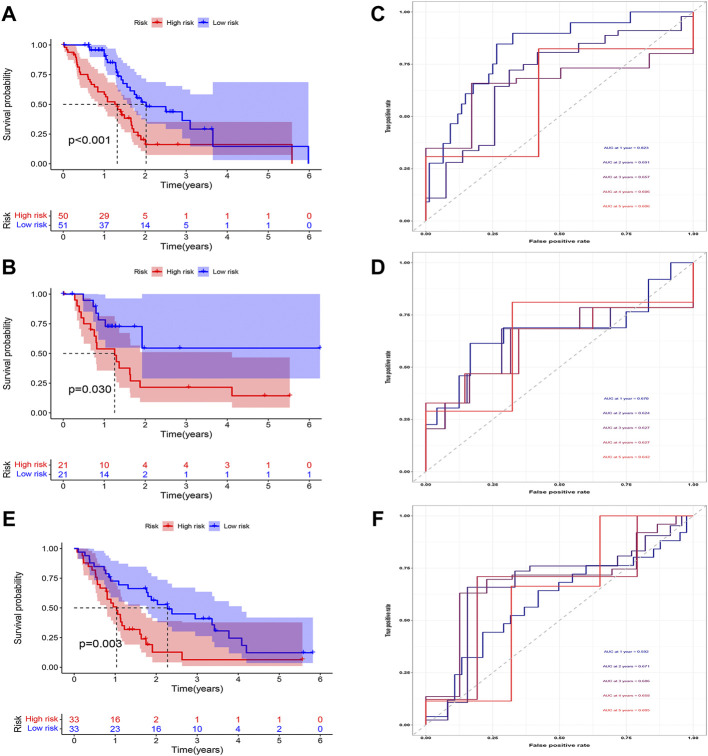
Evaluation of CDRG-based prognostic model. **(A,B)** We found that patients in the high-risk group had a poorer prognosis in both the training **(A)** and test **(B)** cohorts (*p* < 0.05). **(C,D)** We found that the AUC in both cohorts was basically between 0.6 and 0.7. **(E,F)** In the validation cohort, the high-risk patients had a worse prognosis and the AUC was between 0.6 and 0.7.

We then analyzed the distribution of gene expression and patient survival in the models between the high—and low-risk groups in training and test cohorts (Figures 3A,B). We found that with the increase in risk value, the proportion of BC patients who died increased (Figures 3C,D). Moreover, we found that genes EPS8, CASC8, TATDN1, NT5E, and LDHA were highly expressed in the low-risk group(Figures 3E,F). Survival analysis showed a significantly poorer prognosis for patients in the high-risk group (Figures 4A,B). At 1, 2, 3, 4, and 5 years, the AUC values for the training cohort were 0.823, 0.691, 0.657, 0.606, and 0.606, respectively (Figure 4C). At 1, 2, 3, 4, and 5 years, the AUC values for the test cohort were 0.670, 0.624, 0.627, 0.627, and 0.642, respectively (Figure 4D).

In the validation cohort, the survival analysis revealed the model successfully stratified the patients (Figure 4E). At 1, 2, 3, 4, and 5 years, the AUC values were 0.694, 0.729, 0.658, 0.620, and 0.825, respectively (Figure 4F).

### Enrichment analysis

**FIGURE 5:**
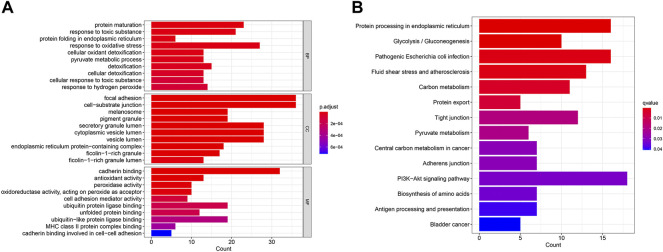
GO enrichment analysis **(A)** and KEGG enrichment analysis **(B)** of CDRG. **(A)** The results of GO enrichment analysis showed that these genes were mainly related to protein processing and maturation **(B)** The results of the KEGG enrichment analysis showed that these genes were mainly related to glycolysis and sugar metabolism and protein processing, and transport.

Then, we performed the enrichment analysis. The results of GO enrichment analysis showed that these genes were mainly related to protein processing and maturation (Figure 5A). The results of the KEGG enrichment analysis showed that these genes were mainly related to glycolysis and sugar metabolism and protein processing, and transport (Figure 5B).

### Immunoassay analysis

**FIGURE 6:**
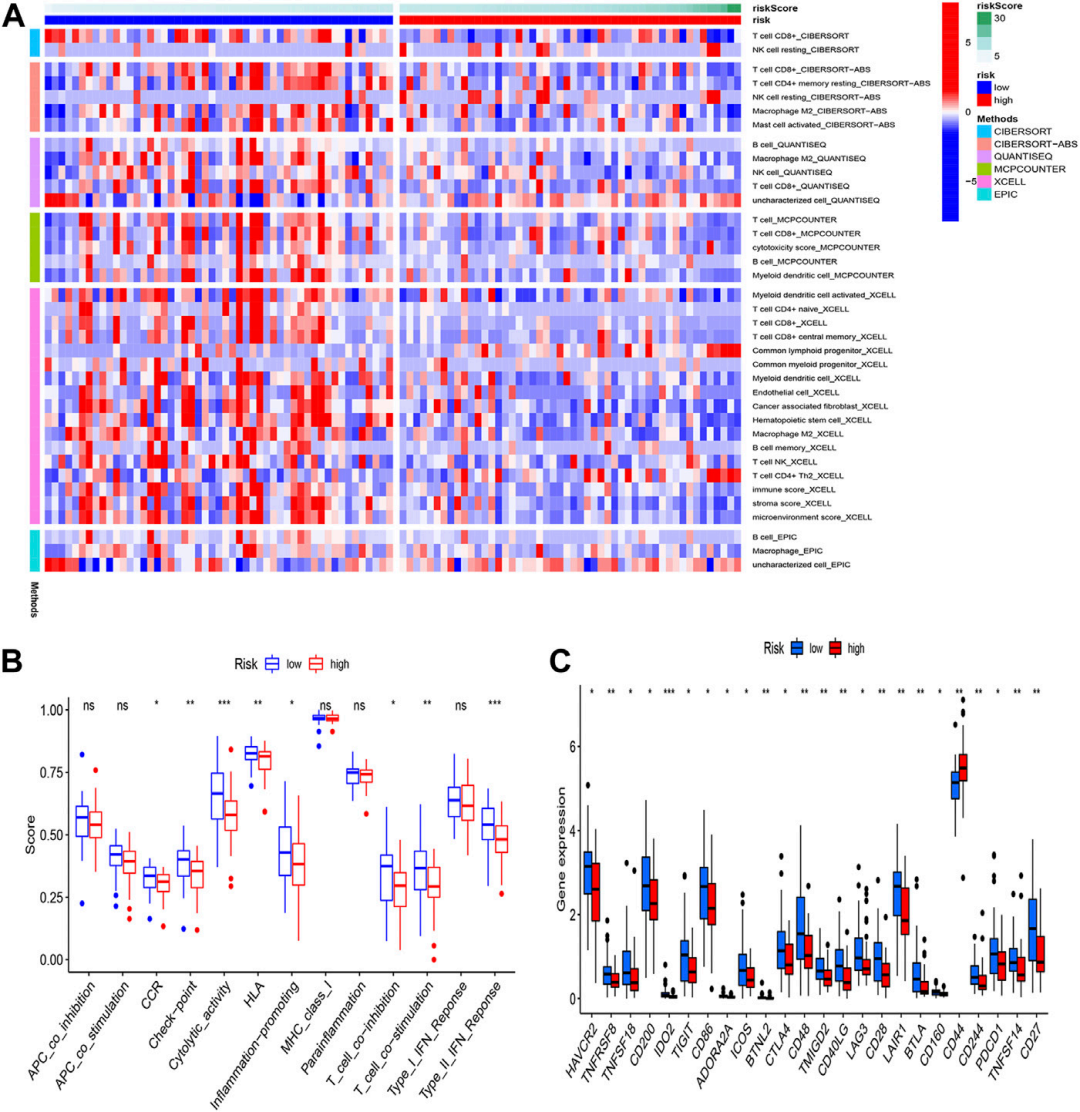
Immunoassay analysis. **(A)** Immune cell infiltration distribution. The distribution of immune cells was significantly different between the high- and low-risk groups. **(B)** Immune-related functions. **(C)** The expression of immune checkpoint-related genes.

In tumor development, the immunological microenvironment is critical. Immunocorrelation analysis showed Myeloid dendritic cell activated, T cell CD4^+^ naive, T cell CD8^+^, T cell CD8^+^ central memory, Common lymphoid progenitor, Myeloid dendritic cell, Endothelial cell, Cancer associated fibroblast, Macrophage M2, B cell memory were significantly related to risk score (Figure 6A). To further understand the differences in immune microenvironments to guide immunotherapy, the immunological function of high-risk and low-risk populations was discussed. Figures 6B,C showed that between the two groups there were significant differences in immune function and the expression of immunological checkpoint genes.

### Identify copper-dependent genes associated with prognosis

After matching transcriptomic data from TCGA and the copper-dependent genes, we matched the copper-dependent gene expression data with survival data and performed independent prognostic analysis, resulting in only the LIPT1 with prognostic significance.

### Identification of the CIC

**FIGURE 7:**
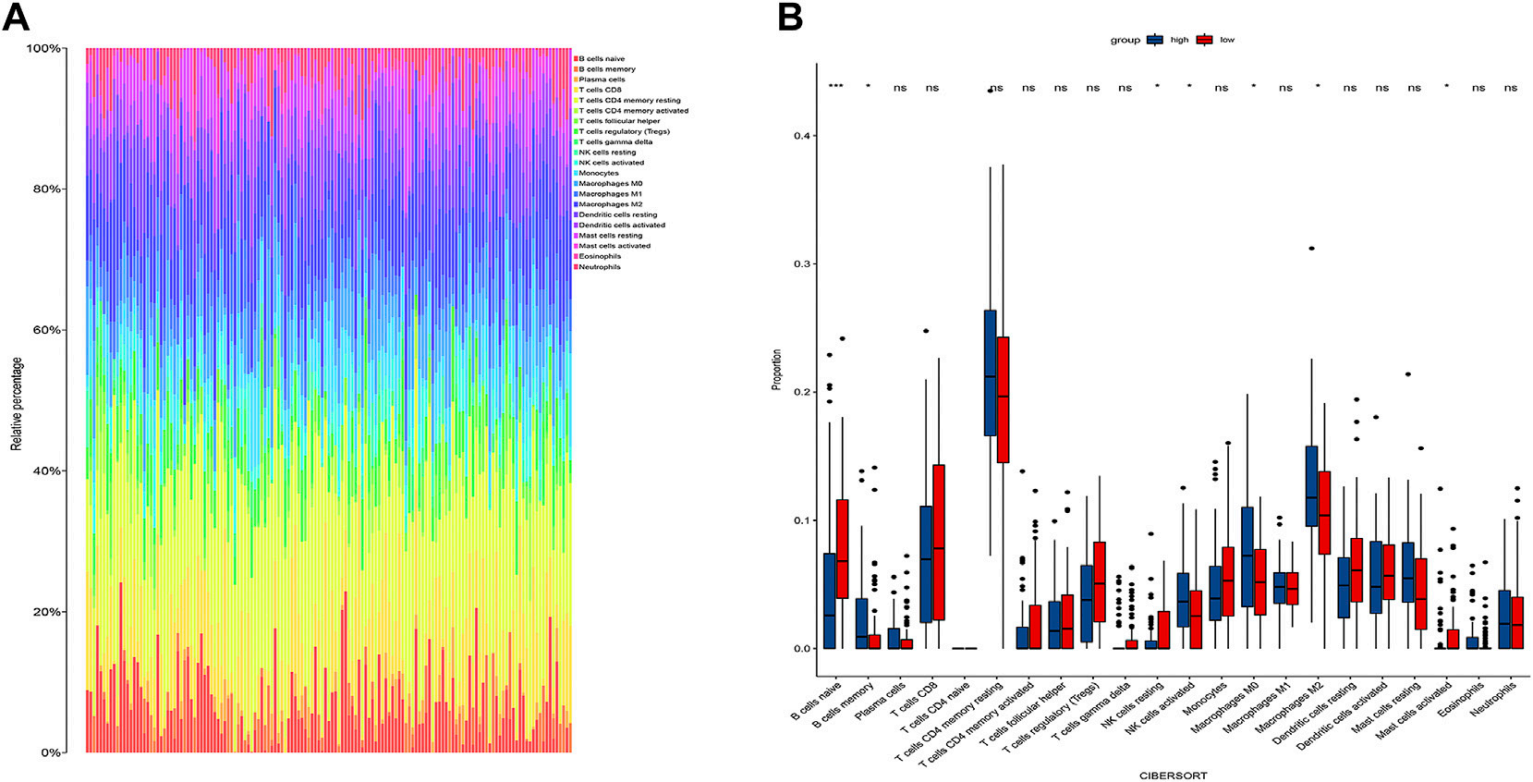
Immune infiltration analysis. **(A)** The proportion of different immune cells in tumors. **(B)** Differences in immune cells between high and low-risk groups. We found differences in B cells, NK cells, and Macrophages between high and low-risk groups. We then performed a LASSO regression analysis and T cells regulatory Tregs, Macrophages.M2, Mast.cells.activated, and Eosinophils were screened out. We retained Macrophages.M2.

We obtained 22 immune cells by CIBERSORT analysis (Figures 7A,B). Only the immune cells linked to CDRG were kept. B cells, NK cells, and Macrophages were significantly different between high-risk and low-risk groups. These cells were identified as CDRG-associated immune-infiltrating cells. We then matched immune-infiltrating cell data with survival data for further screening by LASSO regression analysis. T cells regulatory Tregs, Macrophages.M2, Mast.cells.activated, and Eosinophils were screened out. We retained Macrophages.M2.

### Construction and evaluation of the combined prognostic model

**FIGURE 8:**
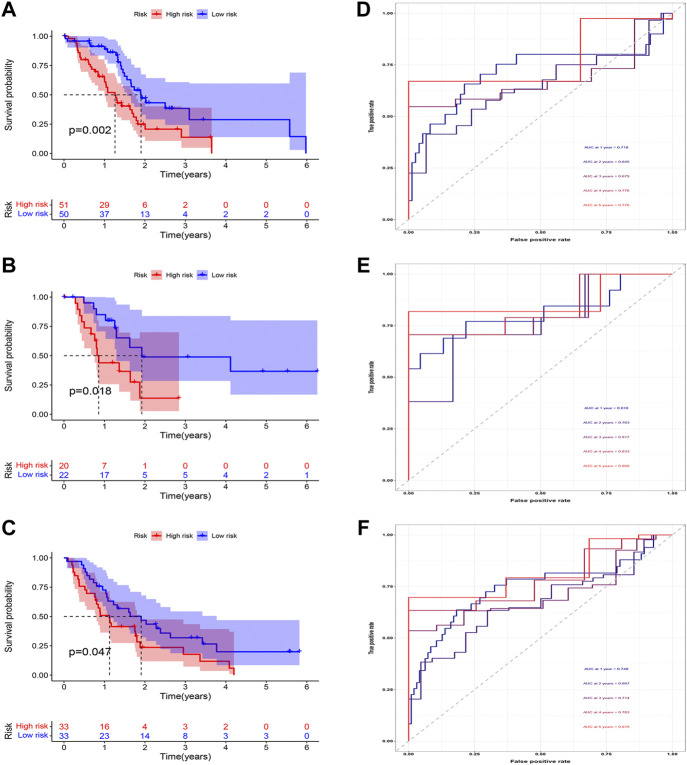
Evaluation of the combined prognostic model. **(A–C)** In training, test, and validation cohorts, The high-risk patients had a worse prognosis **(D–F)** The AUC in t training, test, and validation cohorts was basically between 0.7 and 0.9.

According to the expression of LIPT1, the risk score, and the immune infiltration of Macrophages.M2, a combined model of prognosis prediction was established. The combined model was calculated as follows: combined score = risk score*0.09297–LIPT1*1.29283–Macrophages.M2*2.51248. Survival analysis showed a significantly poorer prognosis for patients in the high-risk group (Figures 8A–C). At 1, 2, 3, 4, and 5 years, the AUC for the training cohort were 0.718, 0.645, 0.679, 0.776, and 0.776, respectively (Figure 8D). At 1, 2, 3, 4, and 5 years, the AUC for the test cohort were 0.810, 0.763, 0.817, 0.833, and 0.868, respectively (Figure 8E). At 1, 2, 3, 4, and 5 years, the AUC for the validation cohort were 0.740, 0.667, 0.714, 0.783, and 0.819, respectively (Figure 8F). The AUC in three cohorts was basically between 0.7 and 0.9, demonstrating that the combined prognostic model was accurate and stable and better than the CDRG-based prognostic model.

### Drug sensitivity analysis

**FIGURE 9:**
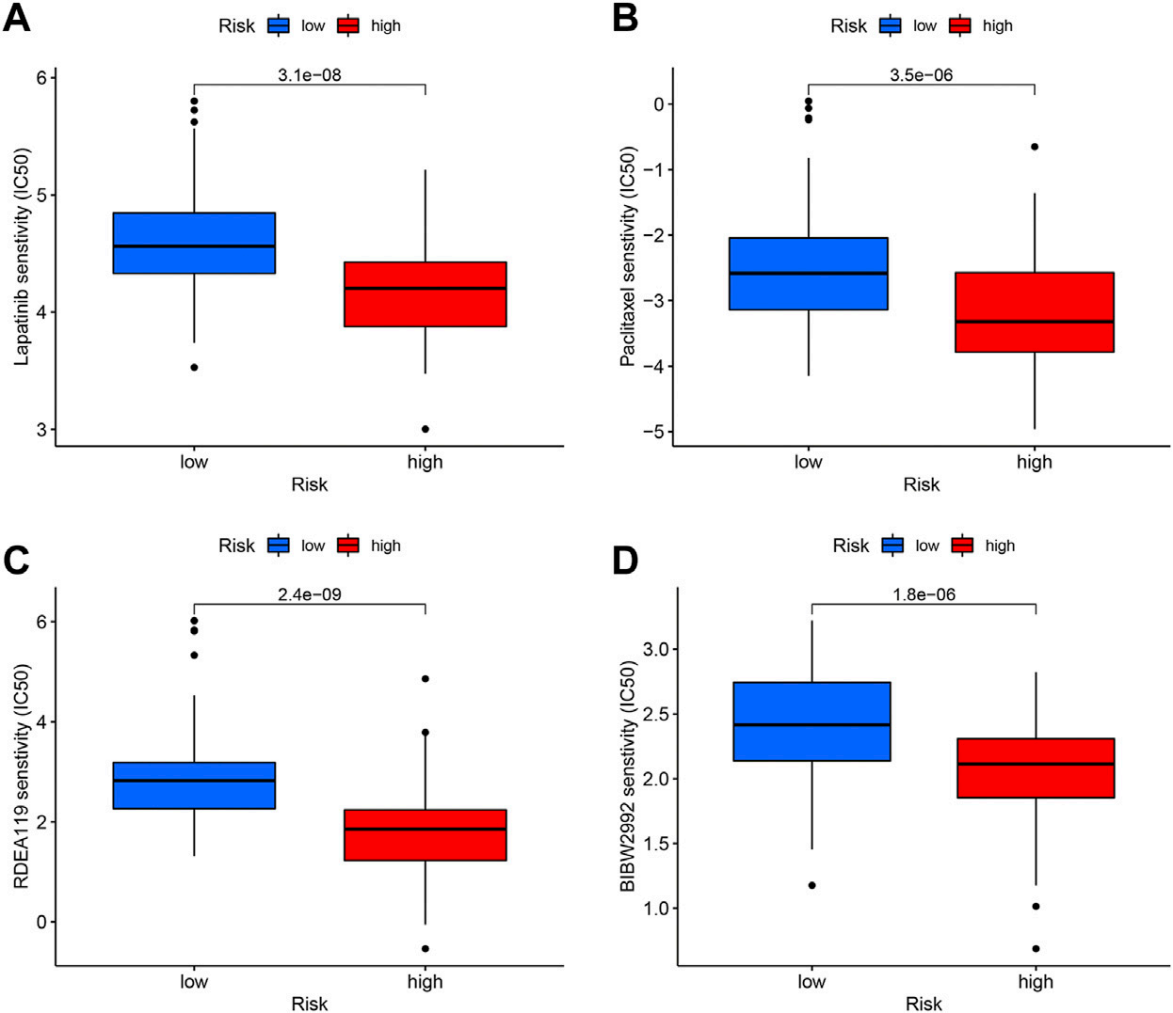
Drug sensitivity analysis. The candidates are Lapatinib **(A)**, Paclitaxel **(B)**, Refametinib **(C)**, and Afatinib **(D)**.

To target treatment, in the high-risk group, drug sensitivity tests were carried out in order to identify medications that were more effective. The results illustrated that Lapatinib, Paclitaxel, Refametinib, and Afatinib had lower IC50, meaning that they were more susceptible to the medications (Figure 9).

## Discussion

Extensive bioinformatics analysis was performed in this study to investigate the significance of copper-dependent genes, CDRG, and immune cell infiltration in pancreatic cancer. Using the GEO and TCGA datasets, this study effectively builds a combined model based on copper-dependent genes, CDRGs, and immune cell infiltration, which can effectively stratify the risk of pancreatic cancer patients and predict their performance in training cohorts, testing Time to live in the queue and validation queue. Furthermore, we found the combined model’s predictive performance was superior to that of the CDRG-based prognostic model alone. We also confirmed that the roles of CDRGs in the immune microenvironment differ significantly among them, which may provide new predictors for immunotherapy in pancreatic cancer patients. Drug sensitivity analysis identifies more sensitive drugs for high-risk groups, which is valuable for the stratified treatment of pancreatic cancer.

Programmed cell death has received increasing attention in tumor therapy and immune microenvironment research (Wang et al., 2021b; Niu et al., 2022). Copper-dependent death is a newly proposed concept that occurs through the direct binding of copper to fatty acylated components of the Krebs cycle (Wang et al., 2021a). Copper acts as a cofactor for mitochondrial cytochrome c oxidase and meets the energy needs of rapidly dividing cells. Therefore, tumor cells require more copper than non-dividing cells (Lopez et al., 2019). Ge et al. (2022) also showed that more of this metal nutrient, such as copper, is required during tumor development and metastasis. Copper concentrations have been shown to be elevated in serum or tumors of many cancer patients, such as colorectal, breast, gallbladder, or thyroid cancers (Basu et al., 2013; Pavithra et al., 2015; Baltaci et al., 2017; Stepien et al., 2017). Studies have shown that the copper-dependent enzyme Lysyl oxidase-like 2 (LOXL2) can remodel tumor cells and promote tumor cell metastasis (Zhan et al., 2019; Lin et al., 2020; Yun et al., 2022). Safi et al. (2014) suggest the copper signaling axis as a new target for prostate cancer therapy. In addition, Bulatov et al. (2018) reported that a copper-based metal complex, the Isatin-Schiff base-copper (II) complex, can activate p53 protein, inhibit tumor cell proliferation and induce apoptosis. However, studies of genes related to copper dependence in pancreatic cancer are lacking. For the first time, we provide the prognostic features of pancreatic cancer copper-dependent genes and CDRG, which have crucial consequences for pancreatic cancer prognosis.

Six genes in the combined prognostic model have been initially elucidated in the pathogenesis and progression of the disease. LIPT1 encodes an enzyme involved in mitochondrial lipoate synthesis (Habarou et al., 2017). Chen et al. (2021) found that LIPT1 is an important survival-related gene in prostate cancer. Eps8 has been originally discovered to be a substrate for EGFR kinase activity, enhancing EGF responsiveness (Luo et al., 2021). Eps8 enhances the ability of cancer cells to migrate (Chen et al., 2010; Chu et al., 2012). Studies had confirmed that CASC8 was a tumor susceptibility gene (Cui et al., 2018). The study by Yu et al. (2019b) showed that TATDN1 upregulates NOVA1 expression by adsorbing microRNA-140–3p and promotes the proliferative potential of breast cancer cells. NT5E encodes CD73, a key enzyme for AMP hydrolysis in adenosine synthesis (Ghalamfarsa et al., 2019). Alam et al. (2022) built a 13-gene prognostic model to evaluate the prognosis of lung adenocarcinoma, in which NT5E is a key molecule. LDHA is a gene encoding a key glycolytic enzyme (Tang et al., 2022). *Reyna-Hernández et al.* found increased expression of LDAH in invasive cervical cancer (Reyna-Hernández et al., 2022). Our research, which combined these six genes to create a predictive model, could help us better understand tumor cells.

Immunotherapy has a significant effect on many malignant tumors (Motzer et al., 2015; Robert et al., 2015; Sharma et al., 2016). However, Checkpoint blockade has little effect on pancreatic cancer (Royal et al., 2010; Brahmer et al., 2012). Whole-cell therapeutic vaccines have similarly failed to show any effect in late-stage trials (Le et al., 2019). Pancreatic cancer is one of the most immunotolerant tumor types (Bear et al., 2020). Although advances in the pancreatic cancer genome and immune landscape have facilitated the development of targeted therapies, they are only available for a small proportion of pancreatic cancer patients (Nevala-Plagemann et al., 2020). Ultimately, the 5-years overall survival rate for pancreatic cancer patients is less than 10%, highlighting the need for alternative treatment options (Vincent et al., 2011). Our study found that immune cell infiltration and immune checkpoint gene expression were significantly different between high-risk and low-risk groups, which will provide a reference for guiding immunotherapy for pancreatic cancer.

For immune checkpoint genes that did not show significant differences, we think it might be due to cellular communication between tumor cells and immune cells (Liu et al., 2021). The complex immune microenvironment of pancreatic cancer also makes single-agent immunotherapy for pancreatic cancer often unsuccessful (Wu et al., 2019). Understanding the complex interactions of tumor cells with the tumor stroma and the use of targeted drugs in combination with immunomodulatory therapy has shown promising results (Goel et al., 2017; Schaer et al., 2018). Inhibition of the tumor microenvironment combined with immune checkpoint inhibitor therapy can promote effective tumor control (Knudsen et al., 2021).

The study by Gajewski et al. (2013) indicated that cancer must evade anti-tumor immune responses in order to grow gradually. Tumor immune evasion is identified as a characteristic of tumor progression (Batlle and Massagué, 2019). The main immunosuppressive cells in the tumor microenvironment are macrophages, and their infiltration is associated with poor prognosis (Malla et al., 2022). Yan et al. (2022) found that inhibition of Macrophages.M2 polarization inhibited cervical cancer progression. In our study, we explored the difference of Macrophages.M2 infiltration between high-risk and low-risk groups, which had implications for our further targeted therapy in pancreatic cancer.

Drug-resistant treatment is a major challenge in the current treatment of pancreatic cancer (Hennig et al., 2022). Resistance to chemotherapy drugs is also a major cause of poor prognosis in pancreatic cancer patients (Zhu et al., 2022). Our study selected drug candidates relevant to prognostic models, which had implications for our further pancreatic cancer treatment.

However, our study has some limitations. Firstly, the research data comes from the TCGA and GEO public databases. In the future, *in vivo* or *in vitro* basic experiments will be performed to confirm our findings, and we will further refine them in the future. Secondly, Our study is a retrospective study based on previous data. Prospective clinical validation is needed henceforth, which we will improve in the future. Finally, The copper-dependent-related genes defined in this study require further experimental validation.

This is the first combined prognostic model to our knowledge to be constructed using copper-dependent genes, copper-dependent-related genes, and immune cell infiltration profiles. It provides information for the study of programmed death in pancreatic cancer and contributes to the treatment of pancreatic cancer patients.

## Conclusion

Based on copper-dependent genes, copper-dependent-related genes, and immune cell infiltration profiles, the combined prognostic model was built for pancreatic cancer. We can accurately estimate the prognosis and immunological microenvironment of pancreatic cancer patients using this model. In addition, Our findings might lead to new approaches to pancreatic cancer therapy.

## Data Availability

The original contributions presented in the study are included in the article/Supplementary Material, further inquiries can be directed to the corresponding author.
