# Supplementary material for: The combined prognostic model of copper-dependent to predict the prognosis of pancreatic cancer
Source: Front Genet. 2022 Aug 10;13:978988. doi: 10.3389/fgene.2022.978988 (PMC9399350; doi:10.3389/fgene.2022.978988)
Supplement: Supplementary file 1 [file Table1.DOCX]

Supplementary Material

## Supplementary Figure S1


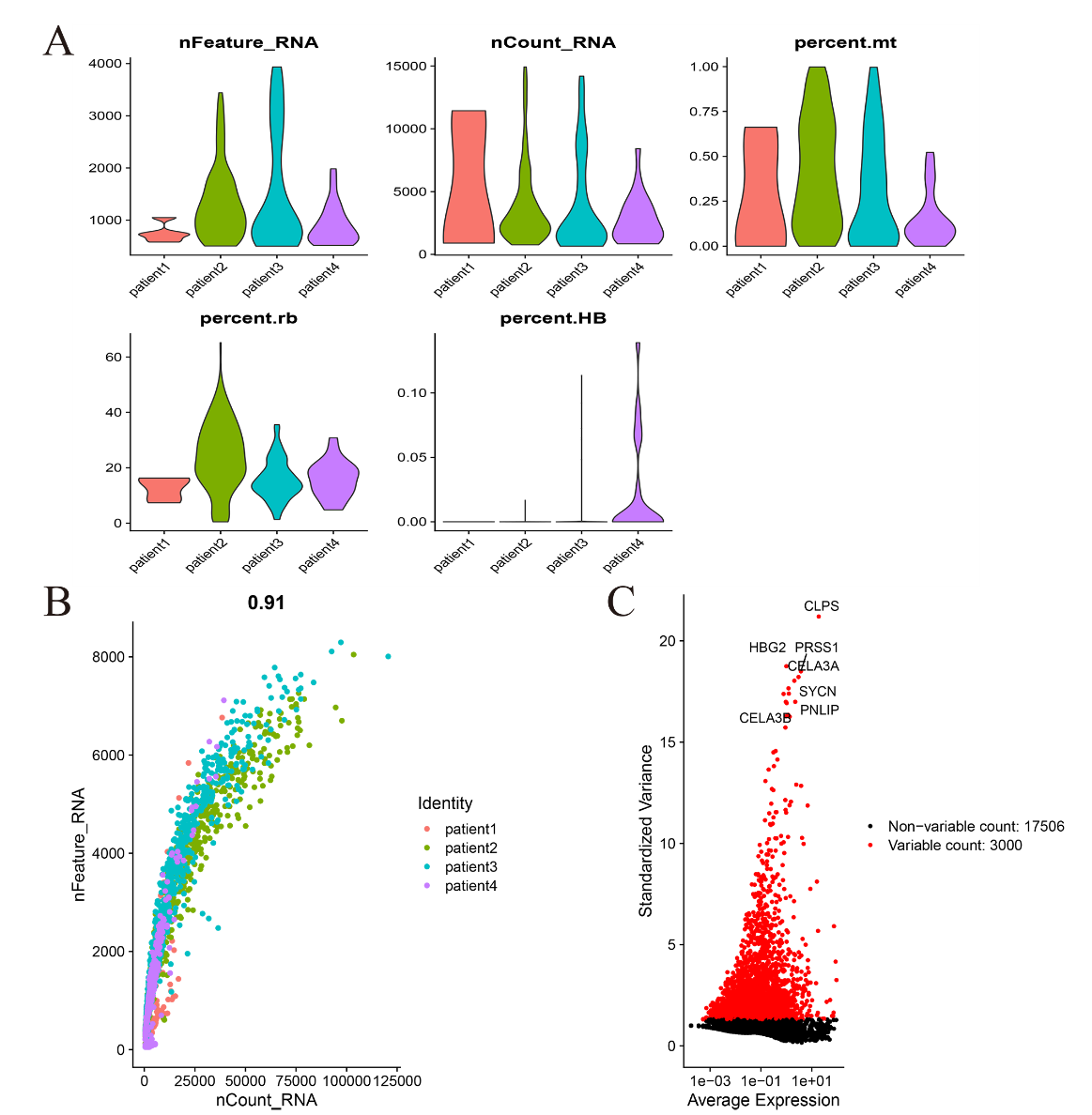


**Supplementary Figure S1**

Quality Control. (A) The amount of gene expression per cell. The gene expression levels of each cell in the 4 samples ranged from 500 to 4000, with a relatively uniform distribution. At the same time, we found that the percentage of mitochondrial genes was less than 1%, and the percentage of erythrocyte genes was basically less than 0.1%. (B) The cells were distributed evenly among the 4 samples. With a correlation coefficient of 0.91, the number of genes and their expression levels are positively correlated. (C) From all genes, we chose 3000 hypervariable genes, which were highlighted in red. We also marked the top 10 genes.
