# Supplementary figures and images for: The combined prognostic model of copper-dependent to predict the prognosis of pancreatic cancer
Source: Front Genet. 2022 Aug 10;13:978988. doi: 10.3389/fgene.2022.978988 (PMC9399350; doi:10.3389/fgene.2022.978988)

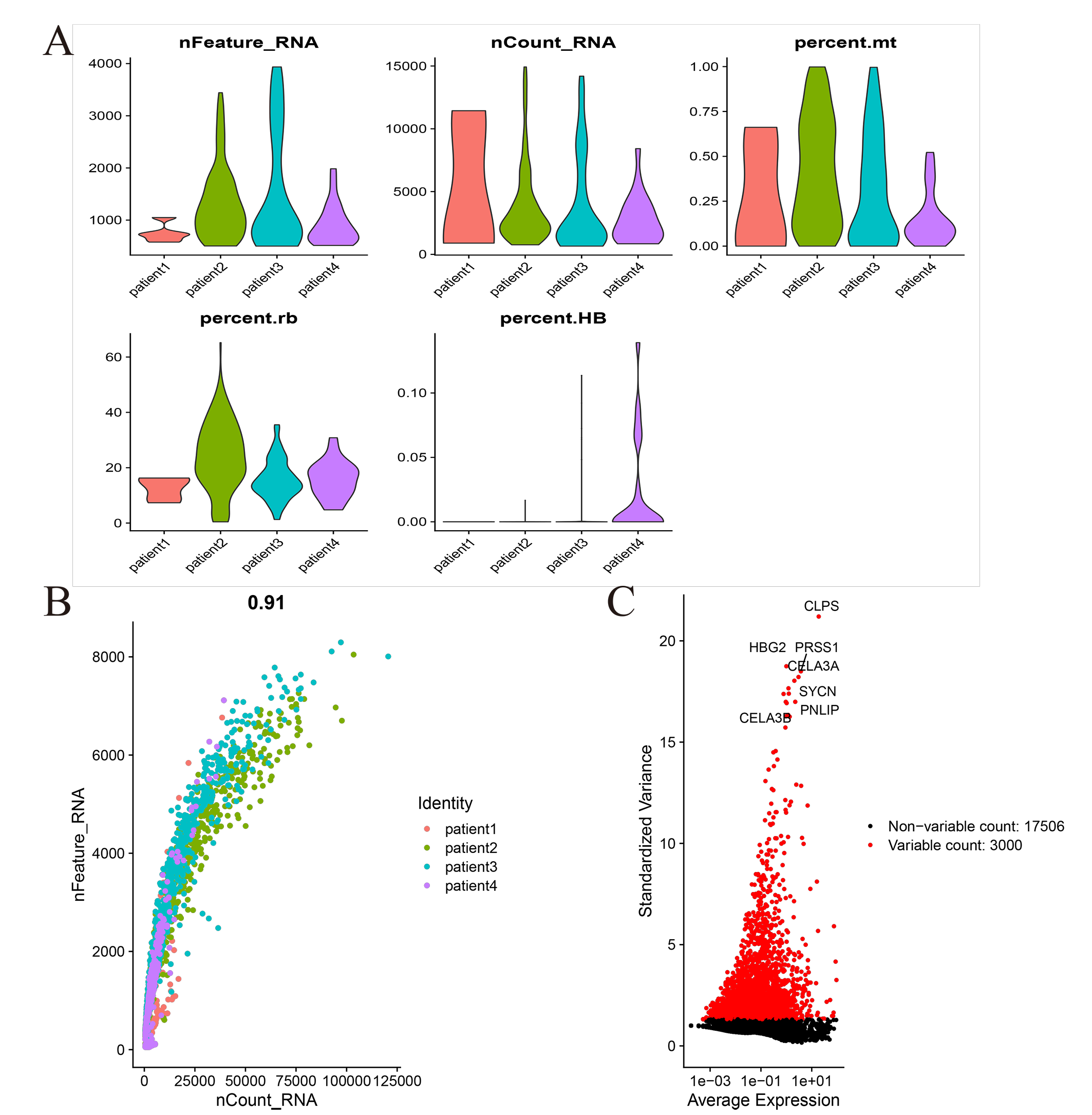

Supplement: Supplementary file 2 [file Image1.TIF]
